# An aggressive vertebral hemangioma in pregnancy: a case report

**DOI:** 10.1186/1752-1947-8-207

**Published:** 2014-06-18

**Authors:** Ouafae Slimani, Sofia Jayi, Fatimazahra Fdili Alaoui, Hakima Bouguern, Hekmat Chaara, Ghizlane Fikri, Siham Alaoui Rachidi, Nadia Sqalli Houssaini, Mariam Himmich, Moulay Abdelilah Melhouf

**Affiliations:** 1 Department of Gynecology Obstetric II, Teaching Hospital Hassan II, Fez, Morocco; 2 Department of Radiology, Teaching Hospital Hassan II, Fez, Morocco; 3 Department of Neurosurgery, Teaching Hospital Hassan II, Fez, Morocco

**Keywords:** Antepartum treatment, Pregnancy, Spinal cord compression, Vertebral hemangioma

## Abstract

**Introduction:**

Pregnancy-related compressive myelopathy secondary to vertebral hemangioma is a rare occurrence and its treatment antepartum is rare.

**Case presentation:**

A 19-year-old North African woman in her 38th week of pregnancy presented with paraplegia that progressed within 2 days after a rapidly progressive weakness of her lower limbs. Magnetic resonance imaging studies showed compression of her spinal cord in front of the fourth thoracic vertebra for suspected tuberculous spondylitis. A Caesarean section was done followed by corpectomy with a bone graft because we intraoperatively discovered a vertebral hemangioma. Pathology showed an aggressive hemangioma.

**Conclusion:**

At any term of pregnancy, extensive neurological involvement which is rapidly progressive due to compression should be considered for immediate decompression.

## Introduction

Vertebral hemangiomas are among the most common benign tumors involving the spine, usually presenting as an incidental finding on imaging (magnetic resonance imaging (MRI) or even on plain radiography) [1]. Pregnancy, because of several changes, is a recognized risk factor coinciding with the development of a rapid onset of symptoms from these normally asymptomatic lesions. Less than 1% of vertebral hemangiomas cause neurologic symptoms from spinal cord or nerve root compression; they can lead to serious neurologic deficits if not treated immediately [2]. So, prompt diagnosis is essential in planning optimal therapy and preventing morbidity for both mother and fetus.

We report a case of an aggressive vertebral hemangioma during pregnancy that was treated surgically after Cesarean delivery and we also review the current literature on pregnancy- related vertebral hemangiomas.

## Case presentation

**Figure 1:**
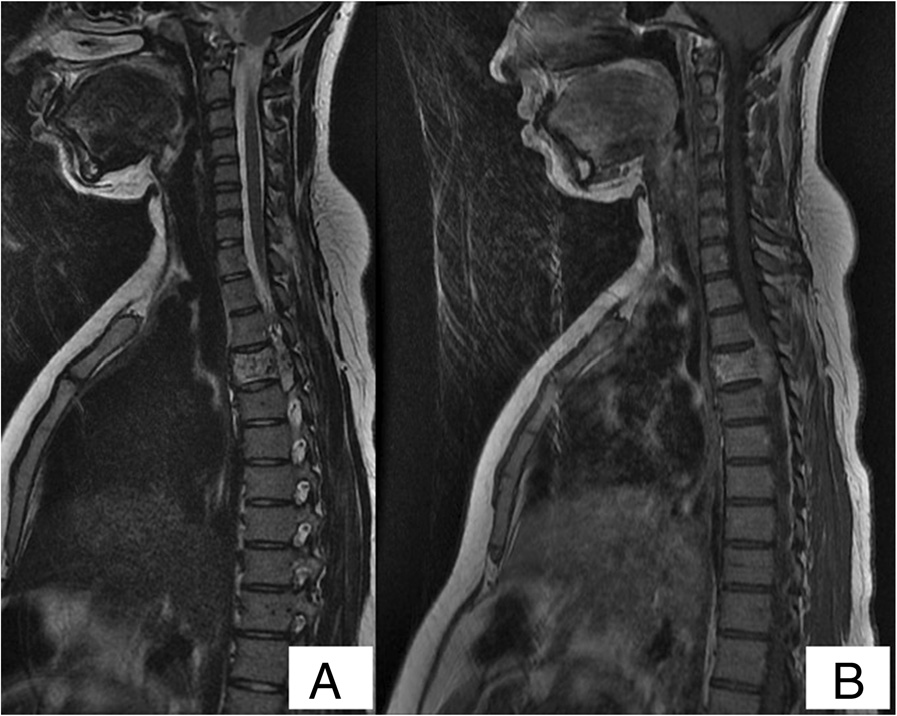
**Lateral tomography radiographs. (A)** T2-weighted magnetic resonance image and. **(B)** T1-weighted magnetic resonance image of the thoracic spine demonstrating the lesion in the T4 vertebra (fourth thoracic vertebra).

A 19-year-old, North African, primiparous woman presented to our emergency room in the 38th week of gestation with a 10-day history of a rapidly progressive weakness of her lower limbs. She mentioned paraplegia progressed within 2 days. She did not have any back pain, incontinence or constitutional symptoms. On examination she was pregnant with uterine height corresponding to gestational age and a positive fetal cardiac activity. There was a spastic paraplegia, without decrease of sensory function of her lower limbs. MRI studies showed compression of her spinal cord located at the T4 level suspicious for tuberculous spondylitis (because of tuberculosis epidemic in our country) (Figure 1). Gadolinium contrast material was not administered because of the patient’s pregnancy. Multidisciplinary discussion among obstetricians, neurosurgeons and intensivists, favored a Caesarean section action, before the intervention for spondylitis that was to be made the day after the Cesarean section.

Thoracotomy was performed via left posterolateral incision with the excision of her left 6th rib, and intraoperative findings suggested a vertebral hemangioma T4 with anterior spinal cord compression. T4 corpectomy as well as excision of most of the calcified epidural mass was done. Her excised rib was put in the corpectomy site in two pieces as a bone graft.

Two units of blood were transfused during the operation. Histopathologic evaluation of the operation specimen showed typical characteristics of capillary hemangioma (aggressive hemangioma).

After the operation, the patient started to move her legs again, and after a month she began to walk with support. Her 3-month follow-up examination revealed a normal neurological examination.

## Discussion

Vertebral hemangiomas are the most common primary spinal neoplasm and are present in 10 to 12% of the population. The majority of patients with vertebral hemangiomas remain asymptomatic, and only 1% are estimated to become symptomatic. Vertebral hemangiomas were first described by Virchow. The typical radiological appearance of vertical striations was described by Perman. The first case of pregnancy-related hemangioma was reported in 1927 by Balado, whereas half of the cases reviewed were published after 1980 [2].

In the literature, 26 cases of symptomatic vertebral hemangioma during pregnancy have been reported [3]. The mean patient age at presentation was 29 years. The great majority of these cases presented during the third trimester of pregnancy. The mean period of gestation at presentation was 7.8 months [3].

Contrary to their usual location in the lower thoracic and lumbar vertebrae in non-pregnant patients, pregnancy-related vertebral hemangiomas occur more frequently in the upper thoracic levels [4].

Our patient also had symptoms in the third trimester of her pregnancy.

Physiologic changes during pregnancy may induce rapid onset symptoms from these normally asymptomatic lesions. By the seventh month of gestation, the gravid uterus begins to compress the vena cava causing obstruction or functional closure [5]. Venous obstruction and increased intra-abdominal pressure cause redistribution and increased blood flow volume through the vertebral venous plexus, resulting in the expansion and growth of previously existing vertebral hemangiomas. This is the most important contributing factor in the clinical manifestation of a pregnancy-induced symptom [6]. The hormonal changes taking place during pregnancy have also been implicated for a growth-promoting effect on an already existing hemangioma of the spine, mainly through structural changes within the vessel wall [7]. Maternal progesterone may increase the venous distensibility. The endothelial growth-promoting effect of estrogen may contribute to an increase in size of a preexisting hemangioma. Relaxin may have an effect on the vessel walls as well [8]. However, immunohistochemical analysis done on vertebral hemangiomas has not been able to demonstrate estrogen and progesterone receptors on tumor tissue and this implicates a hemodynamic rather than a hormonal cause for disease progression.

Usually incidental, asymptomatic, and solitary, these are benign vascular lesions that can give rise to symptoms in rare circumstances. Back pain followed by acutely or subacutely progressing paraplegia and sensory loss were the typical presenting symptoms. More than half of the cases in the literature developed paraplegia within 1 month of onset of back pain [3].

It is postulated that neurological symptoms may be produced by one or more of the following mechanisms: compression fracture of involved vertebrae, sudden hemorrhage into the extradural space, hypertrophy of the posterior cortex of the vertebral body, or enlargement of the lamina and facets as a result of the angiomatous invasion, spinal cord ischemia caused by “steal” and subperiosteal growth of the tumor, and spinal cord compression from extradural mass [9].

On plain radiographs, vertebral hemangiomas exhibit a characteristic vertically striated or irregular honeycomb pattern. On axial computed tomography, they have a “polka-dot” pattern as the vertical trabeculae are imaged in cross-section [2].

As for diagnosis of vertebral hemangiomas, MRI is the first diagnostic choice in pregnancy, because it saves the patient and the fetus from ionizing radiation. According to Pomeranz [10], the characteristic MRI appearance of a vertebral hemangioma is a hyperintense, mottled, or “starburst” signal on T1- and T2-weighted images. After an injection of gadolinium–diethylenetriaminepentaaceticacid, the extradural portion enhances more evidently, compared with that of the vertebral body.

The histological appearance of hemangiomas consists of benign vascular proliferation with normal capillary and venous structure [5]. Two types have been described: cavernous and capillary. The most common type of vertebral hemangioma is the cavernous type, which is characterized by large sinusoidal spaces lined by a single layer of epithelium. The capillary type of vertebral hemangioma differs from the cavernous type only by having smaller vascular channels.

Surgery, radiotherapy, embolization, and various combinations of these have been used in the treatment of symptomatic vertebral hemangiomas. More recently vertebroplasty has been used in the treatment of symptomatic vertebral hemangiomas. Pregnancy is a relative contraindication to radiotherapy. Embolization is not risk free especially during pregnancy, and complications include vascular injury and radiation exposure to the fetus during fluoroscopy [2].

Although endovascular embolization (EE) with particulate agents such as polyvinyl alcohol foam produce dramatic but usually transient remission, Bouchez *et al.*[11] reported the first case of a pregnant patient who had a vertebral hemangioma that was embolized successfully and the patient recovered completely; long-term follow-up is needed to assess the efficacy of this procedure. The second case was reported by Kiroglu *et al.*[3], in which there was no compression fracture, so EE was performed as the first treatment choice, but at the end of 2 uneventful years after EE treatment, the patient’s complaints and pains recurred.

Various surgical approaches and techniques are available for symptomatic vertebral hemangioma, and they need to be tailored for each patient according to the location of the tumor within the vertebra and the patient's symptoms. Percutaneous vertebroplasty (PV), usually with the use of polymethylmethacrylate (PMMA), is a relatively new treatment option for symptomatic vertebral hemangioma. Patients who present with pathologic compression fracture and intractable back pain are good candidates for PV [12]. However, because PV is a palliative treatment and has the risk of PMMA leakage into the spinal canal, it may be contraindicated in patients with epidural hemangioma [12]. Progressive neurologic deficit is the main indication for surgical decompression, especially with rapid onset of symptoms [2]. Anterior corpectomy is potentially curative, and adequate decompression can be achieved in patients with a vertebral body tumor, whereas hemangioma is a highly vascular tumor and intraoperative blood loss may be substantial by corpectomy, and blood transfusion would often be necessary. Laminectomy may afford adequate decompression in selected cases, particularly those with an epidural tumor or with laminar involvement. Laminectomy is apparently not curative when the hemangioma involves the vertebral body. Although laminectomy may be temporarily effective as an emergency decompression procedure, recurrence of symptoms is common, eventually necessitating a definitive treatment for the vertebral body tumor [9]. Although mechanical integrity of the anterior and middle columns was partially restored by vertebroplasty, Castel *et al.*[13] also advocated addition of posterior segmental fixation.

The timing of surgery in cases diagnosed antepartum is controversial, as many patients show spontaneous remission after delivery [14]. However, the symptoms do not disappear completely [9] or often can recur, requiring surgery at a later time.

A management algorithm based on a literature review [2] highlighted two main key aspects in the decision-making process of such scenarios: the week of gestation and the severity of the neurologic impairment. Conservative management, consisting of rest and pain control, was offered to patients approaching the end of their pregnancy (32nd week of gestation or later) or those with only mild or moderate symptoms, whereas any substantial or progressing neurologic deficit was considered an indication for surgical decompression [15].

Indeed, the accepted norm is to delay intervention until a viable fetus can be delivered should premature labor occur [4]. But in the presence of progressive neurological deficits, decompression is essential at the earliest to achieve an optimal outcome especially in those cases that present in the second trimester or early third trimester. The treatment algorithm proposed by Chi *et al.*[2] based on the duration of pregnancy and status of neurological deficits is useful in planning treatment in these cases. Patients at 36 weeks of gestation or later, are observed, if neurological function deteriorates, then one can consider induction of delivery followed by appropriate management of the tumor. Between 32 and 36 weeks of gestation, expectant observation is considered; surgery is reserved for severe cases of paraplegia. For patients in whom gestation is earlier than 32 weeks, prepartum surgical treatment should be considered for those who are severely symptomatic [2].

Our patient underwent a cesarean section and the day after a corpectomy was performed.

A total of 23 cases of pregnancy-related vertebral hemangioma dating back to 1927 were identified. Prepartum surgery was performed in seven patients, postpartum surgery in 12 [5], and no surgery in four [11]. Among patients treated surgically before childbirth, four experienced preterm labor [4]; prepartum surgery was also associated with two maternal deaths [14] and two fetal deaths [4], but all occurred prior to 1985 (data on cases of death have not been explained in the literature).

Among patients treated surgically after childbirth, two improved following delivery [14], two experienced transient improvement followed by decline and persistent deficit, and five deteriorated or had persistent deficits after delivery [5].

Excluding deaths, all patients demonstrated excellent neurological recovery, independent ambulation, and resolution of pain at follow-up review (range 4 months to 5 years). Mild paresthesia and weakness not interfering with activities of daily living were found in two patients [6]. Excluding the two perinatal deaths, there were no reports of adverse effects on the infants, and no adverse effects were noted in the children during the follow-up periods reported.

Revision surgery for worsening symptoms after the initial surgery was performed in two patients [9].

## Conclusions

Pregnancy is a well-recognized condition during which vertebral hemangiomas may become clinically significant. Extensive neurological involvement which is rapidly progressive due to compression should be considered for immediate decompression.

Despite the accumulating information on the management of symptomatic hemangiomas during pregnancy, we found no class I data to support any specific modality of treatment, nor the preferred timing for its application.

## Consent

Written informed consent was obtained from the patient for publication of this case report and any accompanying images. A copy of the written consent is available for review by the Editor-in-Chief of this journal.

## Competing interests

The authors declare that they have no competing interests.

## Authors’ contributions

All the authors participated in the literature search, interpretation of the articles reviewed and analysis of the data and review of the manuscript. All the authors have read and approved the final version of the manuscript.
